# Gapless edge states localized to odd/even layers of AA′-stacked honeycomb multilayers with staggered *AB*-sublattice potentials

**DOI:** 10.1038/s41598-023-44084-9

**Published:** 2023-10-07

**Authors:** Kyu Won Lee, Cheol Eui Lee

**Affiliations:** Department of Physics, Korea University, Seoul, 02841 Republic of Korea

**Keywords:** Materials science, Physics

## Abstract

In honeycomb multilayers with staggered *AB*-sublattice potentials, we predict gapless edge states localized to either of the odd and the even layers for the AA$^{\prime }$ stacking order in which the sublattice-pseudospin polarizations of adjacent layers are antiparallel. Gaps in the projected layer-pseudospin spectrum suppress interlayer hopping between odd and even layers. The layer-valley Chern number corresponding to the edge states was obtained by decomposing the occupied state into two layer-pseudospin sectors by using a projected layer-pseudospin operator. For the AB$^{\prime }$ stacking, the sublattice-pseudospin polarizations of adjacent layers are antiparallel, but the layer-pseudospin spectrum gap closes at the interface of the topologically different states, leading to gapped edge states. For the AA and AB stackings where the sublattice-pseudospin polarizations of the adjacent layers are parallel, the gapless edge states corresponding to quantum valley Hall states are evenly distributed across the layers.

## Introduction

The low-energy physics of graphene that can be described by the two-dimensional Dirac fermions introduces new degrees of freedom derived from the two-dimensional honeycomb structure^1–4^. Sublattice pseudospin arises from two nonequivalent sites per cell of the honeycomb lattice, and the valley degree of freedom arises from the two nonequivalent *K* points in the Brillouin zone^2,3^. The valley degree of freedom has been experimentally confirmed through a spin-valley-dependent optical selection rule^5–7^. Sublattice pseudospin contributes to many interesting properties of graphene, such as the half-integer quantum Hall effect, and has been proposed as a physically measurable real angular momentum^8–10^. The low-energy physics of bilayer graphene that can be described by massive chiral fermions introduces a layer-pseudospin degree of freedom^11^. In bilayer transition metal dichalcogenides (TMD), valley- and layer-dependent spin-orbit splitting suppresses the interlayer hopping so that the spin orientation is locked to the layer pseudospin^4,12,13^. The spin-pseudospin coupling has been confirmed in several experiments and gives rise to the megnetoelectric effects such as electric field-induced spin Zeeman splitting^4,12,13^. It has recently been suggested that direct coupling between the valley and the gate electric field can be enabled by a valley-layer coupling mechanism, where the electronic states in the valley have valley-contrasted layer polarizations^14^.

**Figure 1:**
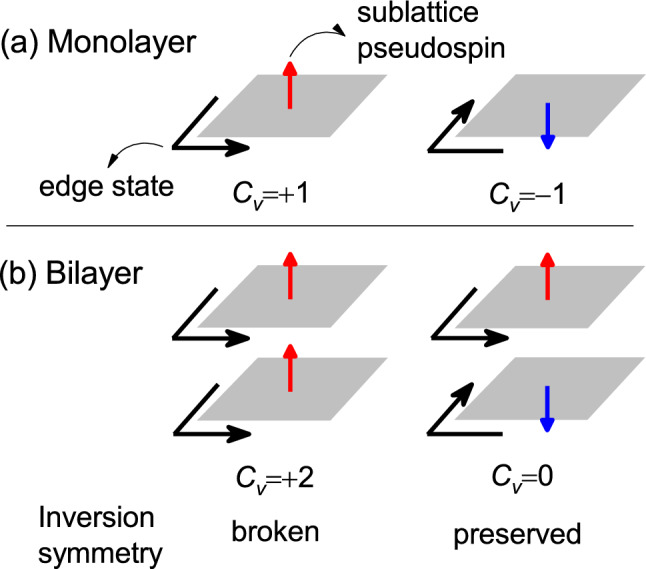
Schematic for layer-valley coupling. (**a**) Two topological domains of a honeycomb monolayer. (**b**) Two stacking types of honeycomb bilayers. Only the edge states corresponding to one of the two valleys are shown. The edge states corresponding to the opposite valley propagate in the opposite direction. Red and blue arrows correspond to the opposite sublattice-pseudospin polarization.

In honeycomb monolayers, a staggered *AB*-sublattice potential ${\Delta }$ can break the inversion symmetry and results in a quantum valley Hall state with a valley-resolved Chern number ${C(\xi )}$=0.5${\xi }$sign(${\Delta }$), where ${\xi =\pm 1}$ is the valley index^15^. The sign(${\Delta }$) can be replaced by the sublattice-pseudospin index ${\tau =\pm 1}$ because the staggered AB-sublattice potential creates a charge imbalance between the two sublattices, resulting in a sublattice-pseudospin polarization. The valley Chern number is defined as the subtraction of the two valley-resolved Chern numbers, ${C_{v}=C(+1)-C(-1)=\tau }$^16^. The sublattice-pseudospin index ${\tau }$ corresponds to two topological domains with ${C_v}$=${\pm 1}$, as shown in Fig. 1a.

**Figure 2:**
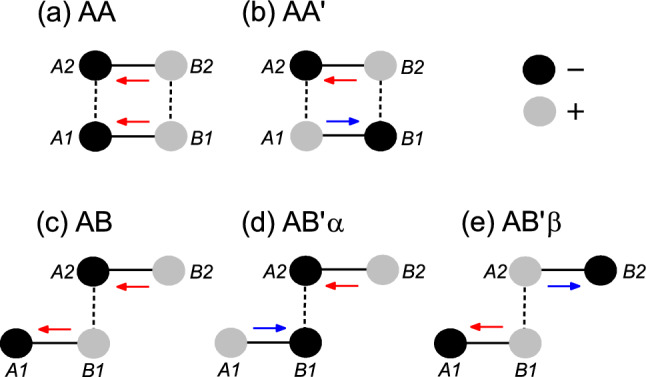
Schematic for high-symmetry stacking orders. Solid and dashed lines correspond to the nearest neighbor hopping within and between layers, respectively. *Ai* and *Bi* indicate *A*- and *B*-sublattices in the *i*-th layer, respectively. Black and grey discs indicate negative and positive on-site potentials, respectively. Red and blue arrows correspond to sublattice-pseudospin polarizations in the opposite directions.

In honeycomb bilayers with staggered *AB*-sublattice potentials, each layer has its own sublattice-pseudospin polarization. The sublattice-pseudospin polarizations of the two layers can be parallel (breaking the inversion symmetry) or antiparallel (preserving the inversion symmetry) depending on the stacking order, as shown in Fig. 1b. Figure 2 shows five high-symmetry stacking orders of honeycomb bilayers with staggered *AB*-sublattice potentials, which can be found in diatomic honeycomb bilayers such as BN, SiC and TMD bilayers^4,7,17,18^. In the AA and AB stackings, sublattice-pseudospin polarizations of the two layers are parallel and the inversion symmetry is broken. In the AA$^{\prime }$ and AB$^{\prime }$ stackings, the sublattice-pseudospin polarizations of the two layers are antiparallel and the inversion symmetry is preserved.

Coupling between different degrees of freedom leads to the expectation of new topological states related to the combined degrees of freedom^16^. Most topological states are characterized by Chern numbers. According to Prodan’s approach, a (pseudo)spin Chern number can be defined if the eigenvalue spectrum of the projected (pseudo)spin operator consists of two isolated sectors with nonzero Chern numbers^19^. Spin Chern insulators can be characterized by the spin Chern number obtained by decomposing the occupied state into two spin sectors using the projected spin operator^20^. Since the occupied state of a honeycomb bilayer can accommodate two pseudospin states^21^, pseudospin Chern numbers related to sublattice- or layer-pseudospins are expected to be obtained following Prodan’s approach. Bilayer configurations generated by folding monolayers with domain walls were used to predict valley Chern states^22^, and bilayers with opposite Rashba-type spin-orbit coupling in adjacent layers have been proposed as building blocks for constructing three-dimensional topological insulators^23^.

In this work, we show that interlayer hopping of gapless edge states can be suppressed by the gap in the projected layer-pseudospin spectrum, leading to gapless edge states localized to either of the odd and the even layers. The layer-valley Chern number corresponding to the edge states was obtained by decomposing the occupied state into two layer-pseudospin sectors. The layer-valley Chern number is a topological invariant unless the band gap or the layer-pseudopsin spectrum gap is closed. At the interface of topologically different states, either the band gap or the layer-pseudospin spectrum gap needs to be closed. We found that in the AA$^{\prime }$ stacking, the layer-pseudospin spectrum has a gap at the interface, suppressing the interlayer hopping of the edge states, and there exist gapless edge states localized to the odd and the even layers, respectively. On the other hand, in the AB$^{\prime }$ stacking, the layer-pseudospin spectrum has no gap at the interface, allowing the interlayer hopping of the edge states, and there exist gapped edge states. In the AA and AB stackings, gapless edge states corresponding to quantum valley Hall states are evenly distributed across the layers.

## Results and discussion

First, we consider noninteracting honeycomb bilayers with staggered *AB*-sublattice potentials. A bilayer with no interlayer interactions can be considered a bilayer in which the separation between the layers is large enough so that the wavefunctions do not overlap. The valley Chern number of the bilayer with no interlayer interactions is given by the sum of the valley Chern numbers of the individual layers, ${C_{v}=\sum _{i=1}^{N=2}{\tau _{i}}}$, where ${\tau _i}$ corresponds to the orientation of the sublattice-pseudospin polarization of the *i*-th layer. For the parallel sublattice-pseudospin polarizations where ${\tau _{i}=\tau }$, ${C_v}$=${\pm }$2. For the antiparallel sublattice-pseudospin polarizations where ${\tau _{i}=(-1)^{i}\tau }$, ${C_v}$=0. Since the quantum valley Hall state can only occur when the inversion symmetry is broken, ${C_v}$ must be zero in the inversion-symmetric bilayers. However, in the absence of interlayer interactions, the individual layers of the inversion-symmetric bilayer is still quantum valley Hall insulators with gapless edge states. Although the valley Chern number is zero, the gapless edge states indicate that the inversion-symmetric bilayer is still topologically nontrivial.

A non-zero Chern number corresponding to the gapless edge states of the inversion-symmetric bilayer can be defined by subtracting the valley Chern numbers of the two layers, ${C_{pv}=\sum _{i=1}^{N=2}{(-1)^{i}\tau _{i}}}$, which can be referred to as the layer-valley Chern number, just like the spin-valley Chern number^16^. Thus, ${C_{pv}}$=0 for the inversion symmetry-broken bilayer and ${C_{pv}}$=${\pm }$2 for the inversion-symmetric bilayer. The gapless edge states corresponding to the layer-valley Chern number can be described by the coupled layer-valley degree of freedom ${\xi \mu }$ defined as the product of the valley index ${\xi =\pm 1}$ and the layer-pseudospin index ${\mu =\pm 1}$, similarly to the coupled spin-valley degree of freedom^24^. If the layer-valley Chern number is not zero, the coupled layer-valley index is locked to the propagation direction of the edge state. In the inversion-symmetric bilayer, backscattering of the edge states requires an inversion of the coupled layer-valley index ${\xi \mu }$. For example, if the valley index ${\xi }$ is fixed as shown in Fig. 1b, backscattering of the edge states requires an inversion of ${\mu }$, which corresponds to the interlayer hopping and is not allowed in the noninteracting bilayer.

Moreover, the definition of ${C_{pv}}$ is independent of an inversion symmetry and can be extended to an arbitrary number *N* of layers. For honeycomb multilayers where ${\tau _{i}=\tau }$, ${C_v}$=${{\pm }N}$ and ${C_{pv}}$=${{\pm }N}$ mod 2. For honeycomb multilayers where ${\tau _{i}=(-1)^{i}\tau }$, ${C_v}$=${{\pm }N}$ mod 2, and ${C_{pv}}$=${{\pm }N}$. If the number of layers is even, only one of the two Chern numbers has a nonzero value. If the number of layers is odd, interestingly, both Chern numbers have nonzero values. For example, in all types of trilayers, we can expect a gapless edge state localized to the layer. Of course, this prediction is trivial for noninteracting multilayers where all the gapless edge states are already localized to individual layers. For interacting mulatilayers, this prediction should be verified.

In the presence of interlayer interactions, the superposition of edge states propagating in opposite directions can lead to gapped edge states in the inversion-symmetric bilayer. Below, however, we show that interlayer hopping of the edge states can be suppressed by the gap in the projected layer-pseudospin spectrum by using a tight binding (TB) model. And we show that the layer-valley Chern numbers obtained by decomposing the occupied state into two layer-pseudospin sectors by using the projected layer-pseudospin operator and subtracting the valley Chern numbers of the two sectors agree with those predicted in the noninteracting case.

**Figure 3:**
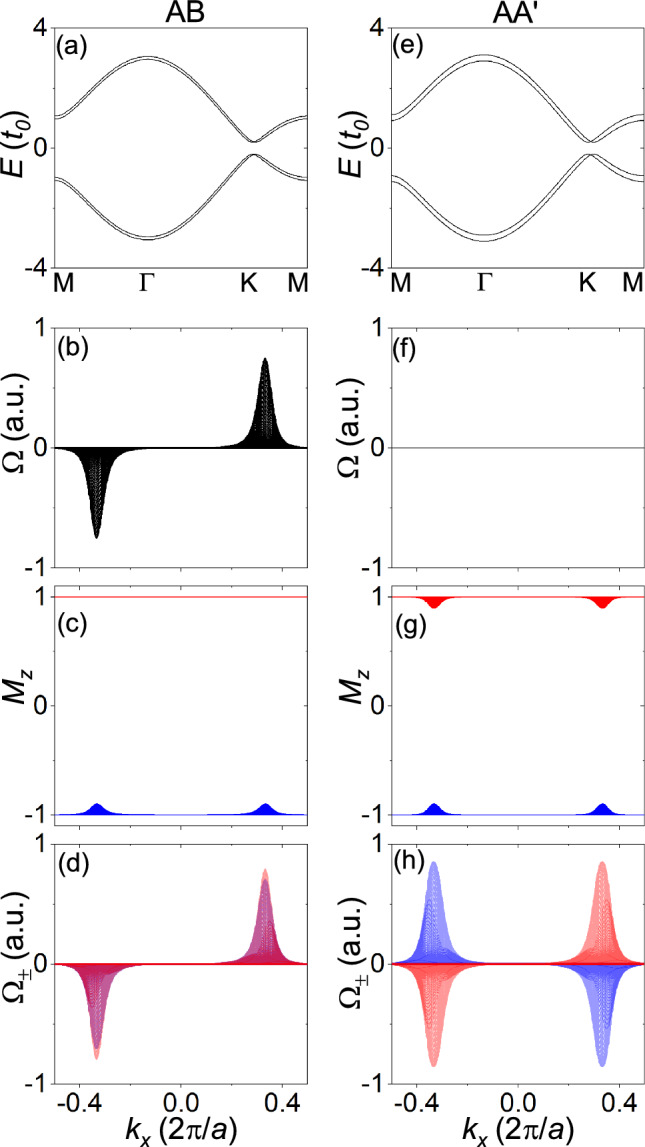
TB model calculations for honeycomb bilayers. The first and second columns correspond to the AB and AA$^{\prime }$ stackings, respectively. (**a**, **e**) Band structures along high-symmetry points. (**b**, **f**) Berry curvatures, (**c**, **g**) layer-pseudospin spectra, and (**d**, **h**) layer-pseudospin-resolved Berry curvatures, projected on the ${k_x}$ axis. The red and blue colours correspond to the two layer-pseudospin sectors.

Figure 3 shows the TB model calculations for honeycomb bilayers for ${\Delta =0.2t_o}$ (see “Methods” section for calculation details). The first and second columns correspond to the AB and AA$^{\prime }$ stackings, respectively. As shown in Fig. 3a,e, both the AB and AA$^{\prime }$ stackings have band gaps at the *K* point of the Brillouin zone. Inversion symmetry is broken in the AB stacking and preserved in the AA$^{\prime }$ stacking. As shown in Fig. 3b,f, the Berry curvature has peaks of opposite signs at the opposite valleys in the AB stacking, while the Berry curvature is zero over the entire Brillouin zone in the AA$^{\prime }$ stacking. Therefore, the valley Chern number obtained by integrating the Berry curvature is nonzero for the AB stacking and zero for the AA$^{\prime }$ stacking. Figure 3c,g shows the (projected) layer-pseudospin spectrum obtained by diagonalizing the projected layer-pseudospin operator. Since the layer-pseudospin spectrum has gaps, the Berry curvature can be obtained separately in the two layer-pseudospin sectors. Figure 3d,h show the Berry curvature in the two layer-pseudospin sectors. The Berry curvatures of the two sectors have the same signs in the AB stacking and the opposite signs in the AA$^{\prime }$ stacking. Therefore, the layer-valley Chern number obtained by subtracting the valley Chern numbers of the two sectors is zero for the AB stacking and nonzero for the AA$^{\prime }$ stacking.

**Figure 4:**
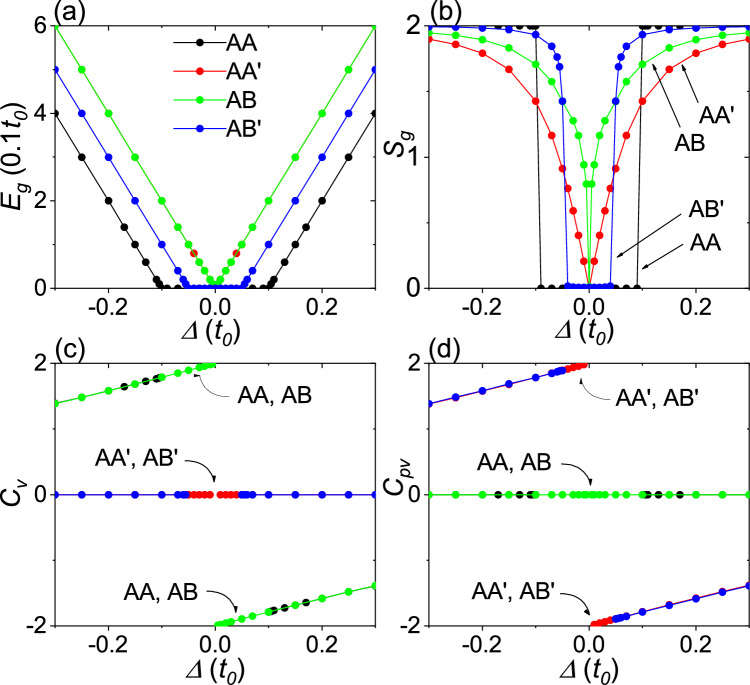
TB model calculations for honeycomb bilayers. (**a**) Band gap ${E_g}$, (**b**) minimal gap ${S_g}$ in the layer-pseudospin spectrum, (**c**) valley Chern number ${C_v}$, and (**d**) layer-valley Chern number ${C_{pv}}$ as a function of the sublattice potential ${\Delta }$. The black, red, green, and blue colours correspond to the AA, AA$^{\prime }$, AB, and AB$^{\prime }$ stackings, respectively.

Figure 4 shows the band gap, the minimal gap in the layer-pseudospin spectrum, the valley Chern number, and the layer-valley Chern number obtained from the TB model calculations for the AA, AA$^{\prime }$, AB, and AB$^{\prime }$ stackings as a function of ${\Delta }$. As shown in Fig. 4a, the band gap ${E_{g}=2|\Delta |}$ opens in both the AA$^{\prime }$ and AB stackings. In the AA stacking, the band gap ${E_{g}=2(|\Delta |-t_{1})}$ opens when ${|\Delta |-t_{1}>0}$. In the AB$^{\prime }$ stacking, the band gap ${E_{g}=2|\Delta |-t_{1}}$ opens when ${2|\Delta |-t_{1}>0}$. The band gap is accompanied by the layer-pseudospin spectrum gap ${S_{g}}$, as shown in Fig. 4b. In the AA$^{\prime }$ stacking, the layer-pseudospin spectrum gap has a simple form, ${S_{g}=2|\Delta |/\sqrt{\Delta ^{2}+t_{1}^{2}}}$, indicating that the layer-pseudospin spectrum gap opens concurrently with the band gap. As shown in Fig. 4c, the valley Chern number is ${C_{v}={\pm }2}$ for the AA and AB stackings, and zero for the AA$^{\prime }$ and AB$^{\prime }$ stackings. As shown in Fig. 4d, the layer-valley Chern number is ${C_{pv}=\pm 2}$ for the AA$^{\prime }$ and AB$^{\prime }$ stackings, and zero for the AA and AB stackings. The Chern numbers ${C_v}$ and ${C_{pv}}$ are consistent with those predicted in the noninteracting bilayers. The gradual deviation of ${C_{v}}$ and ${C_{pv}}$ from integer values as ${\Delta }$ increases can be attributed to the broadening and overlapping of the Berry curvature peaks centered on the opposite valleys^25,26^.

**Figure 5:**
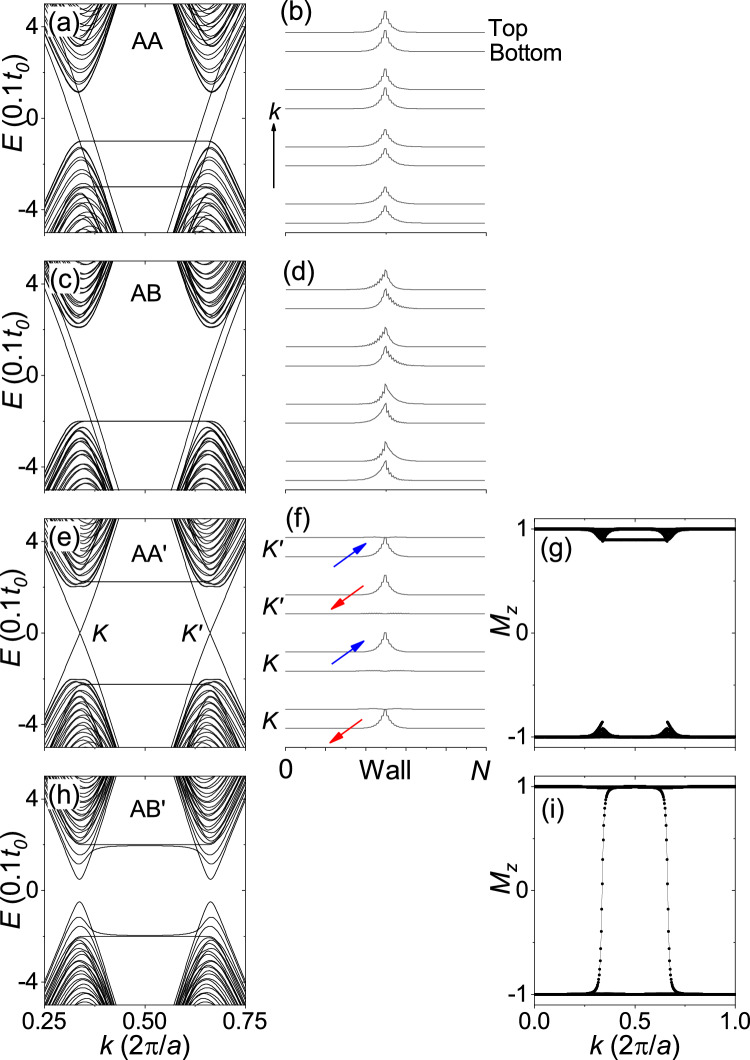
TB model calculations for zigzag-edge bilayer ribbons. 80-ZNR with a topological domain wall in the middle of the ribbon. The first, second, and third columns correspond to the band structure, probability density ${|\Psi |^2}$ at ${E=0}$, and layer-pseudospin spectrum, respectively. The first through fourth rows correspond to the AA, AB, AA$^{\prime }$, and AB$^{\prime }$ stackings, respectively. In (**e**, **f**), *K* and ${K^{\prime }}$ correspond to the two valleys. In (**f**), red and blue arrows indicate the opposite direction of propagation. The broken curve in (**g**) represents the gap in the layer-pseudospin spectrum.

Figure 5 shows the TB model calculations for zigzag-edge bilayer ribbons with a topological domain wall in the middle of the ribbons for ${\Delta =0.2t_o}$ (see “Methods” section for the definition of topological domain walls). Figure 5a,b show the band structure and probability density ${|\Psi |^2}$ at ${E=0}$ for the AA stacking. It can be seen that the gapless edge states fully crossing the bulk band gap are well confined to the domain wall and evenly distributed across the two layers. The propagation direction of the gapless edge state can be determined from the sign of the band velocity (band slope) at ${E=0}$. As shown in Fig. 5a, the band slopes have the same sign at the same valley and the opposite sign at the opposite valley (negative at the *K* valley and positive at the ${K^{\prime }}$ valley), indicating that the gapless edge states belonging to the same valley propagate in the same direction. The gapless edge states belonging to the opposite valley propagate in the opposite direction, so an inversion of the valley is required to reverse the direction of propagation. This indicates that the gapless edge states are prevented from backscattering by the large separation of the valleys, which is typical of the quantum valley Hall states. As shown in Fig. 5c,d, the same is true for the AB stacking.

Figure 5e,f show the band structure and probability density ${|\Psi |^2}$ at ${E=0}$ for the AA$^{\prime }$ stacking, where the gapless edge states that fully cross the bulk band gap are well confined to the domain wall. Interestingly, the gapless edge states are predominantly localized to one of the two layers rather than evenly distributed across the two layers. In Fig. 5e, the gapless edge states belonging to the same valley propagate in the opposite directions, where the valley separation cannot prevent backscattering. As shown in Fig. 5f, the gapless edge states belonging to the same valley are localized to different layers, and the gapless edge states localized to the same layer belong to the opposite valleys. Thus, reversing the propagation direction of an edge state requires an inversion of the valley index or the layer-pseudospin index, indicating that the coupled layer-valley index is locked to the propagation direction. Gapless edge states belonging to the same valley is prevented from backscattering by the gap in the layer-pseudospin spectrum shown in Fig. 5g, because the gap in the layer-pseudospin spectrum suppresses the inversion of the layer-pseudospin index and therefore interlayer hopping of the gapless edge states.

Figure 5h shows the band structure for the AB$^{\prime }$ stacking, where the edge states are gapped. In spin Chern insulators characterized by spin Chern numbers, it has been reported that either the band gap or the gap in the projected spin spectrum needs to close on the sample edge^20^. In Fig. 5i, it can be seen that the gap in the layer-pseudospin spectrum is closed. The absence of gaps in the layer-pseudospin spectrum allows interlayer hopping of the edge states and therefore backscattering of edge states, resulting in the gapped edge states. Both the spin Chern number and the layer-valley Chern number are defined according to Prodan’s approach, which decomposes the occupied state into two (pseudo)spin sectors by using the projected (pseudo)spin operator^19^. The two Chern numbers are topological invariants unless the band gap or the projected (pseudo)spin spectrum gap in the bulk is closed^20^. Except for the fact that we use layer-pseudospins instead of spins to decompose the occupied state, the two Chern numbers are defined in exactly the same way and should have many similarities. The fundamental difference is that each sector in the occupied state has a nonzero valley Chern number in our case and a nonzero (charge) Chern number in the case of a spin Chern insulator^20^. Details of the Chern number calculation can be found in the “Methods” section.

**Figure 6:**
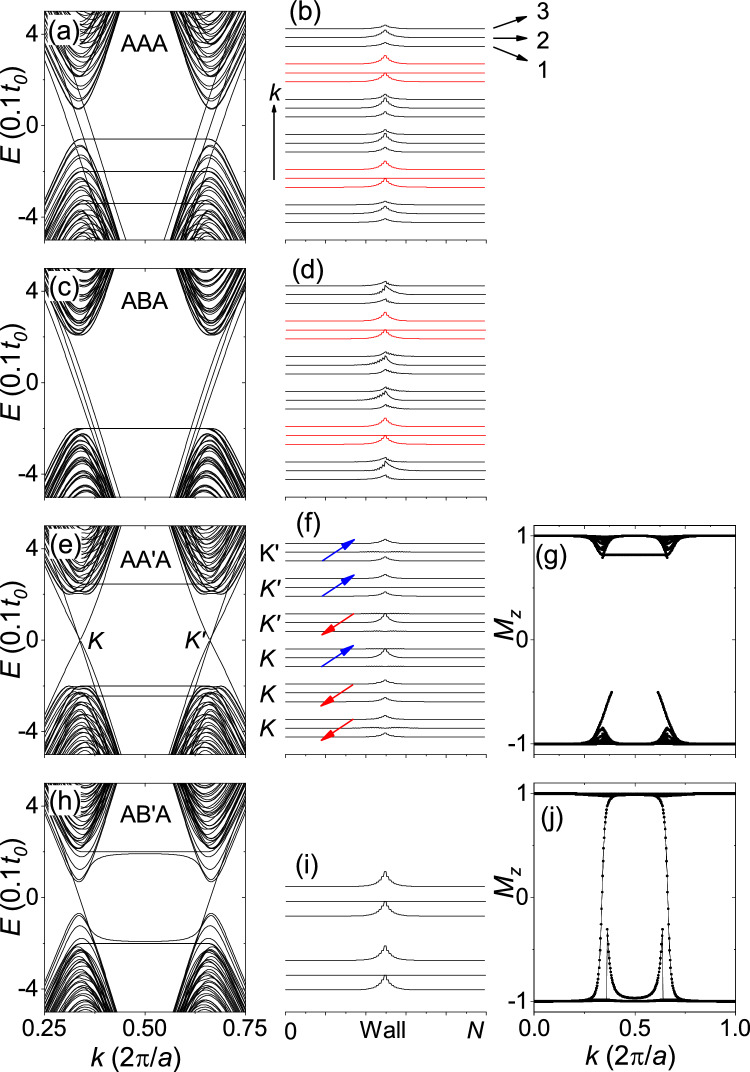
TB model calculations for trilayer 80-ZNR with a domain wall. The first to third columns show the band structure, ${|\Psi |^2}$ at ${E=0}$, and layer-pseudospin spectrum, respectively. The first to fourth rows correspond to the AAA, ABA, AA$^{\prime }$A, and AB$^{\prime }$A stackings, respectively. In (**b**, **d**), the red lines represent gapless edge states predominantly localized to the odd layers. The broken curve in (**g**) represents the gap in the layer-pseudospin spectrum. The same convention as in Fig. 5 was used.

In honeycomb trilayers, ${C_{v}}$ and ${C_{pv}}$ obtained from the TB model calculations are consistent with those predicted in the noninteracting cases. For the AAA and ABA stackings, ${C_{v}={\pm }3}$ and ${C_{pv}=\pm 1}$. For the AA$^{\prime }$A and AB$^{\prime }$A stackings, ${C_{v}={\pm }1}$ and ${C_{pv}=\pm 3}$. Figure 6 shows the TB model calculations for zigzag-edge trilayer ribbons with a topological domain wall in the middle of the ribbons for ${\Delta =0.2t_o}$. As shown in Fig. 6a–d, in the AAA and ABA stackings, since the gapless edge states propagating in opposite directions belong to the opposite valleys, the gapless edge states are prevented from backscattering by the large separation of the valley. However, as shown in Fig. 6b,d, while the other edge states are distributed across the layers, a pair of the edge states propagating in opposite directions (highlighted by red lines) are localized to the odd layers. Since the edge states have the same layer-pseudospin index, inversion of the valley index is the same as inversion of the coupled layer-valley index.

In the AA$^{\prime }$A stacking, as shown in Fig. 6e–g, the gapless edge states are localized to the odd/even layers. Interlayer hopping between the odd and the even layers is suppressed by the gap in the layer-pseudospin spectrum. Backscattering requires an inversion of the valley index if the edge states have the same layer-pseudospin index, and requires an inversion of the layer-pseudospin index if the edge states have the same valley index. In other words, backscattering requires an inversion of the coupled layer-valley index. In the AB$^{\prime }$A stacking, the gap in the layer-pseudospin spectrum is closed, as shown in Fig. 6j, and therefore the edge states should be gapped because interlayer hopping between odd and even layers is not suppressed. However, as shown in Fig. 6h,i, there exist a pair of gapless edge states with opposite valley indices where backscattering is prevented by the valley separation. As shown in Fig. 6i, the gapless edge states are localized to the odd layers with the same layer-pseudospin index, so the opposite valley index corresponds to the opposite layer-valley index. The layer-localized gapless edge states identified in all trilayers are consistent with predictions by the definition of the layer-valley Chern number.

Finally, we briefly discuss experimental validation. In AB-stacked graphene bilayers with stacking domain walls, valley-polarized conducting channels have been experimentally observed^27,28^. In AA$^{\prime }$-stacked MoS$_2$ bilayers under vertical electric field, valley Hall effect has been experimentally confirmed through a spin-valley-dependent optical selection rule^7^. AA$^{\prime }$-stacked honeycomb bilayers have an inversion symmetry in the absence of a vertical electric field and therefore should not have valley Hall effects. Conducting channels along the stacking domain walls of AA$^{\prime }$-stacked bilayers, if found, can be attributed to gapless edge states localized to odd/even layers. Previous studies have shown that electron spin resonance spectroscopy can be an effective tool to detect surface conduction electrons in topological insulators^29,30^.

To summarize, we have shown that honeycomb multilayers with staggered *AB*-sublattice potentials can have topologically nontrivial states characterized by layer-valley Chern numbers. The layer-valley Chern number is obtained by decomposing the occupied state into two layer-pseduspin sectors and subtracting the valley Chern numbers of the two sectors. The gapless edge states corresponding to the layer-valley Chern number are localized to the odd/even layers, and the gap in the layer-pseudospin spectrum can suppress interlayer hopping of edge states between the odd and the even layers. The coupled layer-valley degree of freedom is defined as the product of the layer-pseudospin index and the valley index. The gapless edge states free from backscattering can be well described by the coupled layer-valley degree of freedom locked to the direction of propagation.

## Methods

A single orbital TB model for honeycomb multilayers can be described as^16,25,31^

1 $$\begin{aligned} H=-t_{0}\sum c_{p,i}^{\dagger }c_{q,i} -t_{1}\sum c_{p,i}^{\dagger }c_{q,j} +\sum \Delta _{p,i}c_{p,i}^{\dagger }c_{p,i}. \end{aligned}$$

${c_{p,i}^{\dagger }}$ (${c_{p,i}}$) is a creation (annihilation) operator of an electron at the site *p* (*A* or *B*) in the *i*-th layer. ${t_0}$=1 is the nearest-neighbor hopping energy within a layer. ${t_1}$ is the nearest-neighbor hopping energy between layers and was set to ${t_{1}=0.1t_0}$. ${\Delta _{p,i}}$ is the on-site potential. On the basis of (${\psi _{A1}}$, ${\psi _{B1}}$, ${\psi _{A2}}$, ${\psi _{B2}}$, ...), the on-site potential was set to ${-\Delta (I_{N}\otimes \tau _{z})}$ for the AA and AB stackings, and was set to ${-\Delta (M\otimes \tau _{z})}$ for the AA$^{\prime }$ and AB$^{\prime }$ stackings, each of which corresponds to parallel and aniparallel alignments of subalttice-pseudospin polarizations in the adjacent layers. ${I_N}$ is the ${N{\times }N}$ identity matrix, where *N* is the number of layers. ${\tau _{z}}$ is the Pauli matrix acting on the sublattice index. *M* is an ${N{\times }N}$ diagonal matrix in which ${M_{ij}=(-1)^{i-1}}$ (${i, j=1, 2,..., N}$) if ${i=j}$ and 0 otherwise. In bilayers, *M* equals ${\sigma _{z}}$, which is the Pauli matrix acting on the layer index.

${M{\otimes }I}$ corresponds to the layer-pseudospin, where *I* is the ${2\times 2}$ identity matrix, and the eigenvalues ${\pm 1}$ correspond to the two states in which the electrons are in the odd and the even layers, respectively. Following Prodan’s approach, the occupied states can be decomposed into two layer-pseudospin sectors by using the projected layer-pseudospin operator^19,20,32,33^. Matrix elements of the projected layer-pseudospin operator are given by ${<\varphi _{m}(\textbf{k})|{M{\otimes }I}|\varphi _{n}(\textbf{k})>}$, where ${\varphi _{m}(\textbf{k})}$ and ${\varphi _{n}(\textbf{k})}$ are the wavefunctions for the occupied state with *m* and *n* running over all the occupied states. After diagonalizing the matrix, the eigenvalues ${M_{z}(\textbf{k})}$ give the (projected) layer-pseudospin spectrum, which can be decomposed into two sectors if the spectrum has a gap. The wavefunctions ${\psi _{\pm }}$ in the two layer-pseudospin sectors are given by linear superpositions of the occupied states with the eigenvector of the projected layer-pseudospin operator as the coefficient of the superposition. The layer-pseudospin-resolved Berry curvature can be defined as ${\Omega _{\pm }=\bigtriangledown \times A_{\pm }}$, where ${A_{\pm }= i<\psi _{\pm }\mid \bigtriangledown \mid \psi _{\pm }>}$ is the layer-pseudospin-resolved Berry connection. The valley- and layer-resolved Chern number ${C_{\pm ,\xi }}$ can be obtained by integrating the layer-pseudospin-resolved Berry curvature over the half Brillouin zone. Thus, ${C_{v}=C_{+,K}-C_{+,K^{\prime }}+C_{-,K}-C_{-,K^{\prime }}}$ is the valley Chen number and ${C_{pv}=C_{+,K}-C_{+,K^{\prime }}-C_{-,K}+C_{-,K^{\prime }}}$ is the layer-valley Chern number, where *K* and ${K^{\prime }}$ correspond to two valleys.

A zigzag-edge ribbon, a one-dimensional system periodic in a zigzag direction, is referred to as *n*-ZNR, where the number *n* of the *A*-*B* pairs on a layer in the unit cell corresponds to the ribbon width. A domain wall was introduced by changing the sign of ${\Delta }$ at the middle of the ribbon. In Fig. 2, the AB${_{\alpha }^{\prime }}$ and AB${_{\beta }^{\prime }}$ stackings correspond to the two domains of the AB$^{\prime }$ stacking, which can be obtained by changing the sign of ${\Delta }$.

## Data Availability

The datasets used and/or analysed during the current study available from the corresponding author on reasonable request.
